# Uncertainty increases curiosity, but decreases happiness

**DOI:** 10.1038/s41598-021-93464-6

**Published:** 2021-07-07

**Authors:** Lieke L. F. van Lieshout, Floris P. de Lange, Roshan Cools

**Affiliations:** 1 Donders Institute for Brain, Cognition and Behaviour, Radboud University, P.O. Box 9101, 6500 HB Nijmegen, The Netherlands; 2 Department of Psychiatry, Radboud University Medical Centre, P.O. Box 9101, 6500 HB Nijmegen, The Netherlands

**Keywords:** Human behaviour, Cognitive control, Motivation, Reward

## Abstract

You probably know what kind of things you are curious about, but can you also explain what it feels like to be curious? Previous studies have demonstrated that we are particularly curious when uncertainty is high and when information provides us with a substantial update of what we know. It is unclear, however, whether this drive to seek information (curiosity) is appetitive or aversive. Curiosity might correspond to an appetitive drive elicited by the state of uncertainty, because we like that state, or rather it might correspond to an aversive drive to reduce the state of uncertainty, because we don’t like it. To investigate this, we obtained both subjective valence (happiness) and curiosity ratings from subjects who performed a lottery task that elicits uncertainty-dependent curiosity. We replicated a strong main effect of outcome uncertainty on curiosity: Curiosity increased with outcome uncertainty, irrespective of whether the outcome represented a monetary gain or loss. By contrast, happiness decreased with higher outcome uncertainty. This indicates that people were more curious, but less happy about lotteries with higher outcome uncertainty. These findings raise the hypothesis, to be tested in future work, that curiosity reflects an aversive drive to reduce the unpleasant state of uncertainty.

## Introduction

Curiosity can be defined as a motivation that stimulates exploration and information seeking^1–4^. Indeed, we consume an enormous amount of information from our surroundings. Curiosity towards obtaining that information is generally regarded as intrinsically rewarding and pleasurable^5,6^. Therefore, the state of curiosity is often associated with positive feelings reflecting an appetitive drive towards obtaining novel information.

However, curiosity has also been casted as “an inconsistency or a gap in our knowledge”^7^. This knowledge gap arises when somebody is confronted with a lack of information or uncertainty^8^. Indeed, many (recent) studies have shown that humans and other animals are driven to obtain information to reduce uncertainty about the world around us^9–15^. For instance, we recently showed that participants were more curious when there was more uncertainty about the outcome of a lottery^14–17^, both for lotteries that signal gains (positive information), as well as for lotteries that signal losses (negative information)^14,17^. This was the case even though the information signaled by the outcome was non-instrumental, so that participants could not learn or optimize behavior based on the perceived outcomes.

Taken together, these studies show that curiosity reflects a drive to reduce uncertainty, but what does it feel like to be in this state of uncertainty? Uncertainty can have aversive as well as appetitive properties. We see for example that humans often seek out and enjoy to engage with situations of high uncertainty, e.g. traveling to unknown places, carrying out scientific research with unknown outcomes, or engaging in crossword or sudoku puzzles. However, we also know that generally, humans do not like to be in a state of uncertainty and consider this state to be aversive^18–22^, or even anxiety-evoking^23^. As a consequence, obtaining information and ending the unpleasant state of uncertainty might be rewarding, consistent with an information-as-reward hypothesis^6^. This is supported by opponent-process theory of motivation^24^, which states that rewards (like drugs of abuse or, in this case, information) can have both appetitive and aversive properties, with reward seeking reflecting both a motivation to maximize the presence of reward as well as a motivation to reduce the aversive state elicited by the absence of (e.g. withdrawal from) reward.

The idea that information can be rewarding is supported by findings in macaque monkeys indicating that primary rewards and information share similar behavioral and neurobiological properties^10–12^. Work with human volunteers has also suggested that the induction of curiosity implicates brain regions linked to aversive conditions, such as the anterior cingulate cortex and the anterior insula, while curiosity relief implicates brain areas related to reward^20^.

### The present experiments

In the current set of studies, we aimed to elucidate whether curiosity is an aversive or an appetitive drive by investigating whether the curiosity-triggering state of uncertainty is aversive or appetitive. Using similar lottery tasks as in our previous work^14,15,17^, we independently manipulated the uncertainty and magnitude of expected value of trial outcomes, as well as the sign of the expected value (valence: gain versus loss). Thus, each lottery was associated with more or less uncertain gains or losses. Furthermore, we manipulated whether participants would see the outcome of a lottery at the end of the trial or not in a block-wise fashion. We did so to investigate the effects of knowing that uncertainty would be relieved versus not relieved at the end of a trial. In Experiment 1, participants were asked to indicate how happy they were that the lottery would be played, whereas the participants of Experiment 2 were asked how curious they were about the outcome of the lottery.

We considered two main hypotheses. If the curiosity-triggering state of uncertainty has appetitive properties, then greater curiosity would be accompanied by greater liking. According to this hypothesis, happiness (Experiment 1) and curiosity (Experiment 2) both increase with outcome uncertainty: People would be more curious as well as happier about lotteries with higher outcome uncertainty^14,15,17^. However, if the curiosity-triggering state of uncertainty has aversive properties, then there would be a dissociation between curiosity and liking. Under this hypothesis, people would be more curious about lotteries with higher outcome uncertainty^14,15,17^, while at the same time less happy when lotteries with higher outcome uncertainty are played.

To preview, we demonstrated that curiosity increased with outcome uncertainty for both gains and losses, replicating previous findings^14,15,17^. In contrast, happiness ratings decreased with higher outcome uncertainty. These findings indicate that people are more curious, but less happy when outcome uncertainty is higher. Surprisingly, there was no effect of outcome presentation. Thus, the effects of outcome uncertainty for both experiments did not depend on knowing whether the outcome would be presented and curiosity would or would not be relieved.

## Methods

### Preregistration and data & code availability

Both experiments and their analyses were preregistered on the Open Science Framework (https://osf.io/yd9gw). All data and code used for stimulus presentation and analyses are freely available on the Donders Repository (https://doi.org/10.34973/7b75-0r20).

### Participants

**Experiment 1:** Thirty-seven healthy individuals participated in Experiment 1, involving happiness ratings. Two participants were excluded because they falsely believed they could influence the lottery outcome by means of their happiness ratings. Another participant was excluded due to a technical problem that occurred during the experiment. The final sample of Experiment 1 consisted of thirty-four participants (21 women, age 25.59 ± 6.46, mean ± SD).

**Experiment 2:** Another thirty-six healthy individuals participated in Experiment 2, involving curiosity ratings. Two participants were excluded due to technical problems that occurred during the experiment. The final sample of Experiment 2 consisted of thirty-four participants (24 women, age 23.32 ± 3.34, mean ± SD).

For both experiments, the sample size of N = 34 included participants was chosen to be able to detect a within-subject effect of at least medium size (d ≥ 0.5) with 80% power using a two-tailed one-sample or paired t-test. All participants gave written informed consent according to the declaration of Helsinki prior to participation. The experiments were approved by the local ethics committee (CMO Arnhem-Nijmegen, The Netherlands) under a general ethics approval protocol (“Imaging Human Cognition”, CMO 2014/288) and were conducted in accordance with these guidelines.

### Procedures

**Figure 1:**
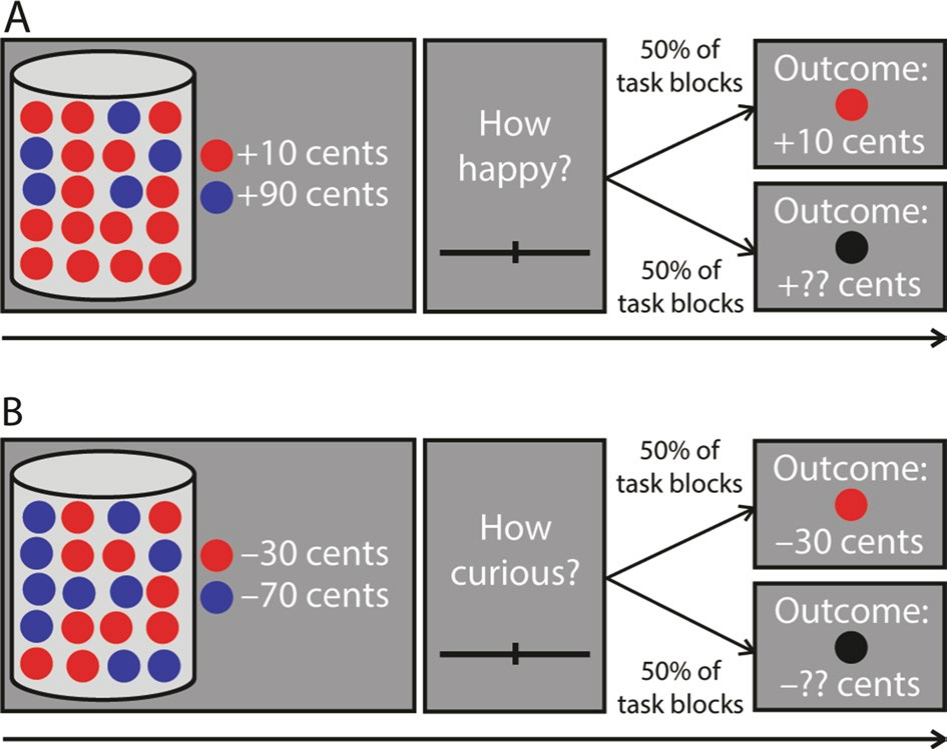
Schematic depiction of the happiness task (a; Experiment 1) and the curiosity task (b; Experiment 2). (**A**) Schematic depiction of a gain trial in the happiness experiment (Experiment 1). Participants saw a screen on which a vase with 20 red and blue marbles was depicted and the monetary values associated with the marbles. These monetary values could either both be positive (in gain trials, depicted here) or both be negative (in loss trials). One marble was selected randomly from the vase and participants would actually gain or lose the money associated with the selected marble. Next, participants indicated how happy they were that the selected lottery would be played on a sliding bar (visual analogue scale). In half of the task blocks, participants would always see the outcome, and in the other task blocks they would never see the outcome. Importantly, participants could not influence which marble would be selected and they were awarded the money associated with the selected marble, regardless of outcome presentation. (**B**) Schematic depiction of a loss trial in the curiosity experiment (Experiment 2). The task was the same as in Experiment 1, but instead of giving a happiness response, the participants had to indicate how curious they were about the outcome. See “Methods” section (*Procedure*) for details on the timing of the experiments.

Both experiments used a similar lottery task (Fig. 1). Each trial started with an image of a vase containing twenty marbles, each of which could be either red or blue. The vases could be configured in three possible ways: (1) 90–10% vases: 18 marbles of one color and 2 marbles of the other color, (2) 75–25% vases: 15 marbles of one color and 5 marbles of the other color, (3) 50–50% vases: 10 marbles of one color and 10 marbles of the other color. Both colors were associated with a monetary value that participants could either gain or lose. These monetary values varied on a trial-by-trial basis between + 10 cents and + 90 in gain trials and between − 90 and − 10 cents in loss trials (both in steps of 10 cents). All combinations of monetary values associated with red and blue marbles were possible, except for combinations of the same monetary values.

We informed the participants that on each trial, one marble would be selected from the vase and that they would gain or lose the money associated with the selected marble. The first screen of each trial depicted the vase, the marbles, and the monetary values associated with the marbles (4000 ms). Next, a blank screen was presented (500 ms), followed by a response screen. On the response screen, participants of Experiment 1 indicated how happy they were that the presented lottery would be played (“How happy are you that this lottery will be played?"), whereas the participants of Experiment 2 indicated how curious they were about the presented lottery (“How curious are you about the outcome of this lottery?”). Participants indicated their happiness (Experiment 1; Fig. 1A) or curiosity (Experiment 2; Fig. 1B) on a sliding bar (visual analogue scale) ranging from 0 to 10. On this scale, a value of 0 means “not happy at all” in Experiment 1 and “not curious at all” in Experiment 2 and a value of 10 means “very happy” in Experiment 1 and “very curious” in Experiment 2. This response screen was presented until the participant gave a response, with a limit of 10 s. The participants used a button box to adjust the position of the bar. The bar would always start in the middle (consistent with a happiness rating or a curiosity rating of 5), and participants could use their right index finger to slide the bar to the left, their right middle finger to slide the bar to the right and their right little finger to confirm and save the response. A blank screen (500 ms), and an outcome screen (2000 ms) followed the response screen. When the outcome was presented, the outcome screen depicted the vase, the marbles and monetary values associated with the marbles again, together with a box in which they saw the colored marble that was selected and the amount of money they gained or lost in that trial. When the outcome was not presented, the participants saw a black marble instead of a colored marble and question marks at the location of the monetary value. This way, the amount of visual input was roughly comparable between presented and not presented outcomes. Participants would always see the outcome of the lottery in half of the task blocks, whereas they would not see the outcome of the lottery in the other half of the task blocks. This manipulation was instructed explicitly before the start of each block and enabled us to investigate whether happiness or curiosity would be a function of knowing that the outcome would or would not presented and uncertainty would be either relieved or not relieved. After a trial ended, there was a blank screen (jittered duration, 1000–2000 ms, uniformly distributed), after which the next trial started.

Importantly, participants were explicitly instructed that they could not influence the outcome of each lottery. The only thing participants had to do was to indicate how happy they were that the presented lottery would be played (Experiment 1) or how curious they were about the outcome of the presented lottery (Experiment 2), given that the outcome would or would not be presented at the end of the trial. In both experiments, participants were told that a marble would be selected on every trial and that they would always gain or lose the money associated with that marble, regardless of outcome presentation. The money they gained or lost in every trial would be summed and the sum of money would be added to or subtracted from the money they earned for participation.

For both experiments, the participants completed a total of 408 trials (204 gain trials and 204 loss trials). Both gain and loss trials were divided in 102 trials during which the outcome was presented and 102 trials during which the outcome was not presented. In turn, each vase configuration was presented on 136 occasions (34 times for each vase configuration). The trials were divided in 8 blocks of 52 trials. During 4 of these blocks, the outcome would always be presented to the participants, whereas the outcome would never be presented in the other 4 blocks. The blocks were pseudo-randomized such that participants would never get the same block type more than 2 times in a row. Within the blocks, the trials were pseudo-randomized, such that participants were never presented with more than six gain trials, six loss trials or six trials with the same vase configuration in a row. After each block, the participants were instructed to take a short break if necessary. The experiment lasted ~ 105 min in total.

### Experimental design

We investigated whether there was a relationship between the main effects of outcome valence (gain/loss), outcome presentation (yes/no), outcome uncertainty, absolute expected value and the happiness ratings (Experiment 1) or the curiosity ratings (Experiment 2). Furthermore, we investigated whether the effects of outcome uncertainty, absolute expected value and outcome presentation (yes /no) on happiness or curiosity differed between the gain and loss trials. This was done by assessing the significance of interaction-effects between outcome uncertainty, absolute expected value, outcome presentation (yes/no) and outcome valence (gain/loss). Likewise, we investigate whether the effects of outcome uncertainty, absolute expected value and outcome valence (gain/loss) on happiness or curiosity differed between trials during which the outcome was presented and trials during which the outcome was not presented. This was done by assessing the significance of the interaction-effects between outcome uncertainty, absolute expected value, outcome valence (gain/loss) and outcome presentation (yes/no).

In order to do so, a value of outcome uncertainty and expected value was calculated for every trial (X) as follows:

1 $$Outcome\;Uncertainty\left( X \right) = \mathop \sum \limits_{{i = 1}}^{2} (x_{i} - Expected\;Value(X))^{2} p_{i}$$

2 $$Expected\;Value\left( X \right) = \mathop \sum \limits_{{i = 1}}^{2} x_{i} p_{i}$$

Here, xi is the monetary value associated with marble (i) and pi the probability that this marble will be drawn. Outcome uncertainty (Eq. 1) reflects the variance or spread of the possible outcomes in trial (X) and expected value (Eq. 2) reflects the mean expected reward for trial (X).

It should be noted that we used a different calculation for outcome uncertainty in one of our previous studies^15^, and we had preregistered to use that calculation of outcome uncertainty in the current manuscript. However, given that both metrics are virtually identical, and variance is a more common measure of uncertainty^25,26^, we decided to calculate outcome uncertainty as variance instead.

It should also be noted that the expected value is always positive in a gain trial and always negative in a loss trial. To be able to compare the effects of expected value between the gain and loss trials, we converted the expected values by taking the absolute values such that − 90 cents in a loss trial would be treated the same as + 90 cents in a gain trial etc. However, the metric of interest here is the effect of reward magnitude (signed expected value) on curiosity, which is reflected in the interaction between outcome valence (gain/loss) and absolute expected value.

#### Statistical analyses

The data were analyzed using a combination of mixed effects modeling using in R (R Core Team, 2013; RRID:SCR_001905; see “Results” section) and classical repeated measure ANOVAs in SPSS (RRID:SCR_002865; see Supplementary Material). This allowed us to verify the robustness of the results and to demonstrate that our conclusions do not depend on the analytical framework employed. The analyses were performed as preregistered, except that instead of running Univariate General Linear Models (GLMs) on the data, we analyzed the data with repeated measures ANOVAs (see Supplementary Material). The motivation for this change in analytical strategy was to be consistent with our previous study using a similar design^14^ and because memory requirements of the univariate GLM model precluded this analysis type within SPSS with the current data set.

#### Analyses using the BRMS package in R

We performed the primary analyses using the brm function of the BRMS package^27^ in R. The analyses were performed as preregistered. The main model of Experiment 1 included “Happiness Rating” (z-scored) as dependent variable, whereas the main model of Experiment 2 included “Curiosity Rating” (z-scored) as dependent variable. The main models included main effects of “outcome valence (gain/loss)”, “outcome presentation (yes/no)”, “outcome uncertainty” and “expected value (absolute)” as fixed effects. Additionally, the main models included interaction effects between “outcome valence (gain/loss)” and “outcome presentation (yes/no)”, between “outcome valence (gain/loss)” and “outcome uncertainty”, between “outcome valence (gain/loss)” and “expected value (absolute)”, between “outcome presentation (yes/no)” and “outcome uncertainty” and between “outcome presentation (yes/no)” and “expected value (absolute)” as fixed effects. The main models included a full random effects structure^28,29^, meaning that a random intercept and random slopes for all effects were included per participant. The predictors for outcome uncertainty and expected value (absolute) were mean centered and scaled. We used the default priors of the brms package (Cauchy priors and LKJ priors for correlation parameters^27^). The main models were fit using four chains with 10,000 iterations each (5000 warm up) and inspected for convergence. Coefficients were deemed statistically significant if the associated 95% posterior credible intervals were non-overlapping with zero.

If the interaction effects between “outcome valence (gain/loss)” and either “outcome presentation (yes/no)”, “outcome uncertainty” or “expected value (absolute)” were significant in the main model, we used the brm function of the BRMS package^27^ to model the gain and loss trials separately. The models were identical to the main model, except that “outcome valence (gain/loss)” as well as its interactions with the other factors were not included. If any of the interaction effects between “outcome presentation (yes/no)” and “outcome uncertainty” or “expected value (absolute)” were significant in these additional models, we modeled the trials in which the outcome was presented and not presented separately.

Similarly, if the interaction effects between “outcome presentation (yes/no)” and either “outcome valence (gain/loss)”, “outcome uncertainty” or “expected value (absolute)” were significant in the main model, we used the brm function of the BRMS package^27^ to model trials in which the outcome was presented and not presented separately. If any of the interaction effects between “outcome valence (yes/no)” and “outcome uncertainty” or “expected value (absolute)” were significant in these additional models, we modeled the gain and loss trials separately.

#### Data visualization

**Figure 2:**
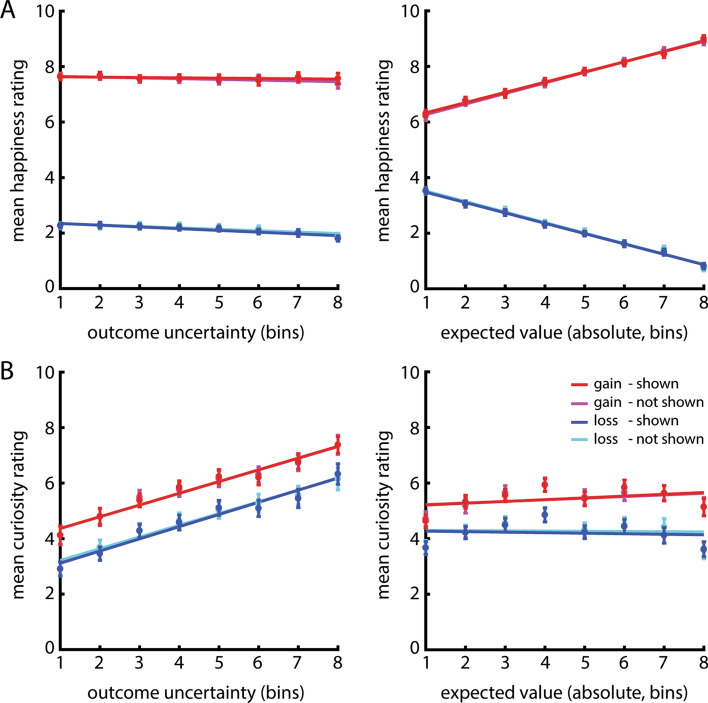
Results of the happiness experiment (a; Experiment 1) and the curiosity experiment (b; Experiment 2). In both panels, the x-axis depicts percentile bins of the values of outcome uncertainty (left) and the absolute values of expected value (right) for both gains (in red; magenta) and losses (in blue; cyan) and for trials in which the outcome was presented (in red; blue) and not presented (in magenta; cyan). The y-axis depicts the mean happiness ratings (**A**) or mean curiosity ratings (**B**) for each percentile of outcome uncertainty and absolute expected value per condition. For other details of the data visualization, see “Methods” section—*Data Visualization.* (**A**) Experiment 1 showed that happiness was higher for gains than for losses. There was a small but significant decrease in happiness with outcome uncertainty, such that participants were happier about lotteries when outcome uncertainty was lower. There was a monotonic increase of happiness with absolute expected value in the gain trials and a monotonic decrease with absolute expected value in loss trials, such that people were happier about trials with higher wins and lower losses. (**B**) Experiment 2 showed that curiosity was higher for gains than for losses. In contrast with Experiment 1, curiosity increased monotonically with outcome uncertainty in the gain and the loss context, such that participants were more curious about the outcome of lotteries when outcome uncertainty was higher. Curiosity increased with absolute expected value in the gain trials, but there was no effect of absolute expected value in the loss trials.

For the data visualization, we divided the levels of outcome uncertainty in eight percentile bins per condition, such that the 1st bin roughly represents the 1/8th of the lowest levels of outcome uncertainty, the 2nd bin the 1/8th–2/8th of the lowest levels, etc. The same was done for the values of absolute expected value. For each bin, a mean happiness score (Experiment 1) or mean curiosity score (Experiment 2) was calculated per participant per condition. Next, these mean scores were averaged across participants and the standard error of the mean (SEM) was calculated for each outcome uncertainty and absolute expected value bin of the gain/loss and outcome presented/not presented conditions separately. Least squares lines illustrate the effects. These results can be seen in Fig. 2A for the happiness ratings and Fig. 2B for the curiosity ratings.

**Figure 3:**
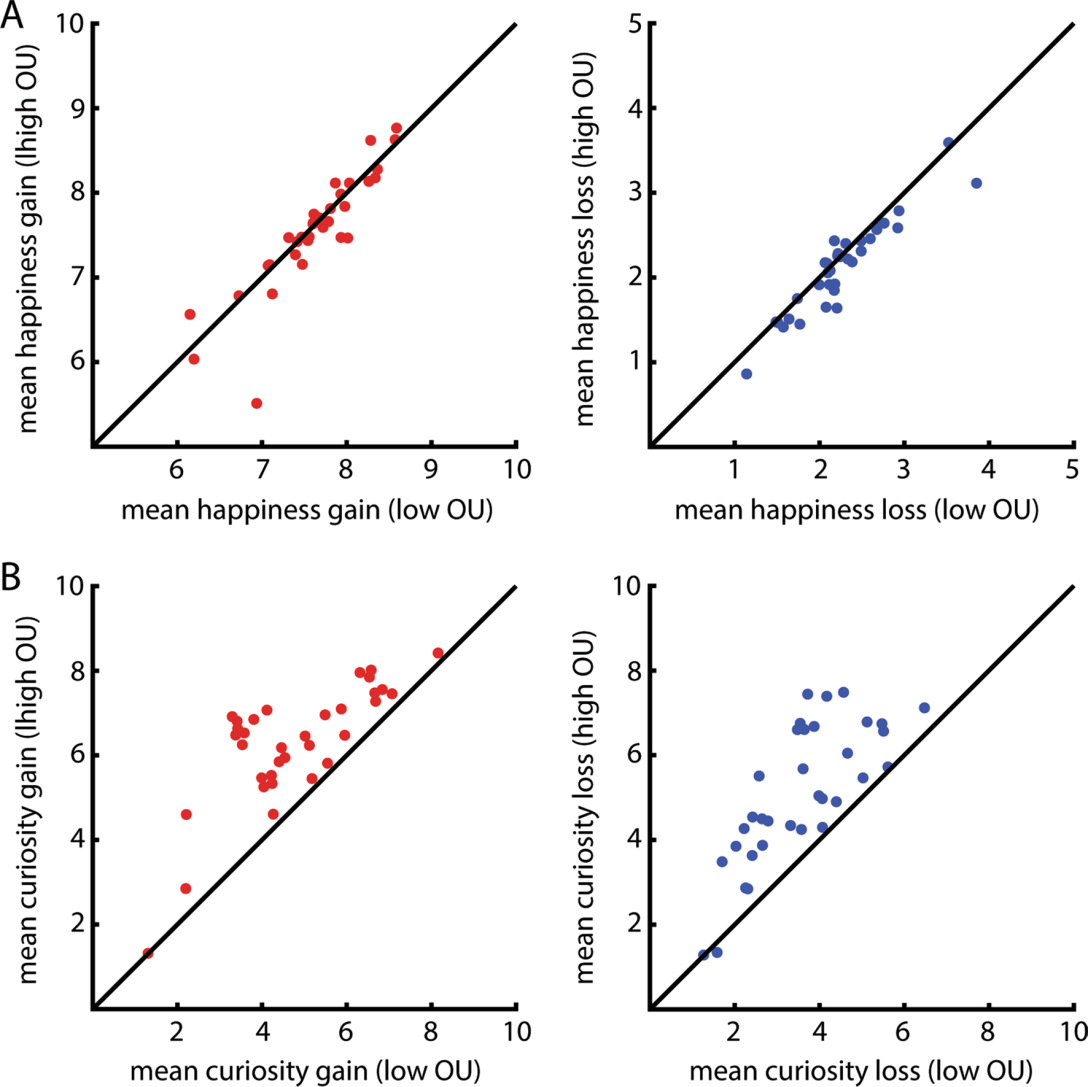
Individual variability in the effects of outcome uncertainty on happiness (a) and curiosity (b). (**A**) Panel A depicts individual data points representing effects of outcome uncertainty on happiness, as a function of outcome valence (left: gains in red, right: losses in blue). In both graphs, the x-axis depicts mean happiness for low outcome uncertainty and the y-axis depicts mean happiness for high outcome uncertainty. Every dot depicts one participant. The majority of the dots are below the diagonal, indicating that participants were happier when lotteries with low compared with high outcome uncertainty were played. This effect was mainly present for the loss trials (blue, right graph) and to a lesser extent for the gain trials (red, left graph). (**B**) Panel B depicts individual data points representing effects of outcome uncertainty on curiosity, as a function of outcome valence (left: gains in red, right: losses in blue). Other conventions are as for panel A. In contrast with panel A, the majority of the dots are above the diagonal, indicating that participants are more curious about the outcome of lotteries with high compared with low outcome uncertainty. This was the case for gains (left, red) as well as for losses (right, blue).

Furthermore, to illustrate to what extent individual participants showed sensitivity to outcome uncertainty, we calculated the mean happiness ratings (Experiment 1, Fig. 3A) and curiosity ratings (Experiment 2, Fig. 3A) for high outcome uncertainty and low outcome uncertainty for the gain (Fig. 3A, B left panels) and loss trials (Fig. 3A, B right panels) separately per participant.

## Results

### Experiment 1

As expected, participants were happier about gain lotteries compared with loss lotteries (Fig. 2A; **BRMS:** 95% CI [0.84, 0.92]). Happiness ratings were not different between blocks in which people would either see or not see the outcome of the lottery (**BRMS:** 95% CI [− 0.011, 0.012]). There was no main effect of absolute expected value (**BRMS:** 95% CI [− 0.010, 0.0067]), but there was an interaction between absolute expected value and outcome valence (**BRMS:** 95% CI [0.25, 0.30]). This interaction was due to participants being happier with higher gains compared with lower gains (**BRMS:** 95% CI [0.25, 0.30]), and with lower losses compared with higher losses (**BRMS:** 95% CI [− 0.30, − 0.24]).

Furthermore, there was a main effect of outcome uncertainty (**BRMS:** 95% CI [− 0.038, − 0.0095]), such that participants were happier about trials with low compared with high outcome uncertainty. We found a small but significant interaction effect between outcome uncertainty and outcome valence when analyzing the data with the BRMS package in R (**BRMS:** 95% CI [0.0002, 0.025]), but this was not the case when analyzing the data with a repeated measures ANOVA (see Supplementary Material). However, when we did analyze the data of the gain and loss trials separately, we found a significant effect of outcome uncertainty on happiness in the loss trials (**BRMS:** 95% CI [− 0.05, − 0.02]), but not in the gain trials (**BRMS:** 95% CI [− 0.04, 0.013]). Therefore, we conclude that the negative effect of outcome uncertainty on happiness is particularly present in the loss trials (Fig. 3A), although we acknowledge that the evidence for differential effects of outcome uncertainty between gain and loss trials is weak.

There were no interaction effects between outcome valence, outcome uncertainty, absolute expected value and outcome presentation (yes/no), indicating that none of the reported effects depended on outcome presentation.

### Experiment 2

Replicating our previous study^14^, participants were more curious about the outcome of gain compared with loss lotteries (Fig. 2B; **BRMS:** 95% CI [0.12, 0.33]). However, curiosity ratings were not different between blocks in which people would either see or not see the outcome of the lottery (**BRMS:** 95% CI [− 0.052, 0.074]). Consistent with our previous study^14^, we found a robust effect of outcome uncertainty, such that participants were more curious about lotteries with higher compared with lower outcome uncertainty (**BRMS:** 95% CI [0.27, 0.43]). There was no interaction between outcome valence and outcome uncertainty (**BRMS:** 95% CI [− 0.024, 0.006]), indicating that this was the case for gain as well as for loss trials (Fig. 3B).

We found a significant interaction between outcome valence and absolute expected value (**BRMS:** 95% CI [0.002, 0.059]). This interaction was due to participants being more curious about high compared with low absolute expected value in gain trials (**BRMS:** 95% CI [0.026, 0.11]), but not in loss trials (**BRMS:** 95% CI [− 0.035, 0.054]). Also in Experiment 2, there were no interaction effects between outcome valence, outcome uncertainty, absolute expected value and outcome presentation (yes/no), indicating that none of the reported effects was dependent on outcome presentation.

## Discussion

We investigated whether curiosity reflects an appetitive or an aversive drive. To this end, we obtained both subjective valence/happiness ratings (Experiment 1) and curiosity ratings (Experiment 2) from subjects who performed a lottery task that elicits uncertainty-dependent curiosity. We found that curiosity robustly increased with outcome uncertainty^14–17^, but that happiness decreased with outcome uncertainty. In other words: people are more curious, but less happy when outcome uncertainty is higher. Surprisingly, the effects of outcome uncertainty on happiness and curiosity did not depend on outcome presentation (that is, knowing whether curiosity would be relieved or not). Together, the current findings indicate that curiosity ratings are higher for states that make people less happy. These findings raise the hypothesis, to be tested in future work, that curiosity represents an aversive drive to reduce the unpleasant state of uncertainty.

Whereas we replicated the finding that curiosity increases with outcome uncertainty for both gains and losses^14,15,17^, we found that happiness *decreased* with outcome uncertainty for gains and losses. Although the negative effect of outcome uncertainty on happiness seems to be smaller than the positive effect of outcome uncertainty on curiosity, both effects are present in the majority of participants (Fig. 3A,B). These distinct effects of outcome uncertainty on happiness and curiosity are in line with findings showing that humans generally consider the state of uncertainty to be aversive^18–22^, and even anxiety-evoking^23^. Thus, uncertainty drives us to seek information in order to reduce the disliked state of ignorance. Although these findings support the hypothesis that curiosity is an aversive drive, they do not discard the information-as-reward hypothesis, according to which information can have appetitive properties. In fact, previous fMRI studies have indicated that self-reported curiosity has been associated with brain activity in the caudate nucleus^30^, the midbrain and the nucleus accumbens^31^. These are structures that are more generally activated by reward anticipation, suggesting that curiosity has appetitive properties. All in all, these findings might be in line with the opponent-process theory of motivation^24^. According to this theory, information seeking is likely driven by a combination of a motivation to reduce the aversive state elicited by the absence of information (uncertainty), as well as by a motivation to maximize the presence of information.

As expected, both curiosity and happiness were greater for gains than for losses. This was expected because our prior work^14,17^, as well as other work^32^, has shown that people exhibit a preference for positive versus negative belief updating. As in the current experiment, obtaining information in those studies was non-instrumental, meaning that there was no way of maximizing rewards or increasing task performance by means of exploration. In these situations, curiosity is higher for gains compared with losses, possibly because it allows the participants to maximize the positive affective state associated with that they have won^33^. Additionally, these effects of outcome valence did not interact with outcome uncertainty. These findings strengthen prior observations that there are multiple mechanisms underlying curiosity^14,17,34,35^, which might operate independent from each other^14,36^. One of these mechanisms is related to an (aversive) drive to reduce uncertainty. The other mechanism is related to processing reward context (savouring): we are both more curious and happier about lotteries containing gains compared with losses.

It should be noted, that our findings seemingly contradict prior work on epistemic curiosity using trivia questions^30,31^. That work suggested that individuals are mostly curious about information of intermediate levels of confidence compared with questions about which they were very confident or not confident at all to already know the answer^30,37^. Other studies using trivia questions even demonstrated that curiosity is strongest when one feels close to filling a knowledge gap^38–40^. These findings contrast with our observation of a linear relationship between uncertainty and curiosity. However, it might reflect that participants in previous work were not curious about questions with highest uncertainty (i.e. lowest confidence), because they were simply not interested in the topics of these questions. In other words, there might be a correlation between one’s disinterest and one’s curiosity about the subject, confounding the relationship between curiosity and uncertainty. Here, this confound was avoided by experimentally manipulating outcome uncertainty in a quantitative and controlled manner.

### Limitations and future directions

Surprisingly, we found no effect of outcome presentation. This indicates that it did not matter for participants whether they would be presented with the outcome or not, and whether uncertainty would be relieved or not. This was the case for both the subjective valence/happiness ratings (Experiment 1) as well as for the curiosity ratings (Experiment 2). These findings are especially surprising given recent findings demonstrating that people experienced less positive affect when the time to close the information gap was longer^41^. Furthermore, under the information-as-reward hypothesis, one would expect that people would be happier when they know that the information gap would be closed by being presented the outcome, compared with situations in which they know that the information gap would not be closed. In both experiments, participants were explicitly instructed that they had to give their happiness or curiosity rating given that the outcome would be presented or not. However, it might be the case that the happiness question was too decoupled from outcome presentation, because the question was focused on the lottery itself (“How happy are you that this lottery will be played?”) instead of on the outcome of the lottery. Another possible explanation is that participants were already cognitively overloaded with processing outcome uncertainty as well as outcome valence and the expected value of a lottery. These values varied on a trial-to-trial basis, whereas outcome presentation varied in a block-wise fashion instead. Perhaps it was easier for people to take the within-block manipulations into account, since they are different every trial and their changes are therefore more salient. Future work might attempt to vary outcome presentation on a trial-to-trial basis, to test the hypothesis that curiosity reflects an aversive drive to reduce the unpleasant state of uncertainty.

The happiness ratings (Experiment 1) and the curiosity ratings (Experiment 2) are obtained from two separate groups of participants. It is therefore not possible to directly compare the effects of outcome uncertainty on happiness with the effects of outcome uncertainty on curiosity. We made the decision for conducting two separate experiments, because we aimed to avoid demand characteristics related to letting participants give more than one rating for similar lotteries (i.e. they could adjust their curiosity rating to their happiness rating or vice versa). Furthermore, we see in Experiment 2 as well as in our previous work^14–17^ that the relationship between outcome uncertainty and curiosity is robust and consistent over participants. It is therefore safe to assume that the participants in the happiness experiment, who overall show that they are less happy when lotteries with higher outcome uncertainty are played, would at the same time be more curious about lotteries with higher outcome uncertainty.

Furthermore, it should be noted that the happiness scale ranged from “not happy at all” to “very happy”. As such, it is not clear whether participants who indicate to be not happy at all, actually consider the lottery to be aversive. It might also indicate that they are simply less happy about these compared with other lotteries, which would be accompanied by more neutral or indifferent feelings about the lottery. Future studies could measure aversion more directly, by ranging the scale from “very aversive” to “very happy”, with the neutral point meaning “not aversive nor happy”. In addition, future studies might leverage the notion that more neutral affect should be accompanied by a reduced autonomic response, measured by pupil dilation^42,43^ or skin conductance^44^.

### Conclusion

These studies advance our understanding of the psychological mechanisms of curiosity, by raising the hypothesis, to be tested in future work, that the curiosity-triggering state of uncertainty reflects an aversive drive. This aversive drive might well go hand in hand with an appetitive drive to maximize information.

## Supplementary Information

Supplementary Information.

## Data Availability

The datasets generated during and/or analyzed during the current study are available in the Donders Repository (https://doi.org/10.34973/7b75-0r20). Both experiments and their analyses were preregistered on the Open Science Framework (https://osf.io/yd9gw).
