# Supplementary material for: Uncertainty increases curiosity, but decreases happiness
Source: Sci Rep. 2021 Jul 7;11:14014. doi: 10.1038/s41598-021-93464-6 (PMC8263743; doi:10.1038/s41598-021-93464-6)
Supplement: Supplementary file 1 — Supplementary Information. [file 41598_2021_93464_MOESM1_ESM.docx]

**Uncertainty increases curiosity, but decreases happiness**

Lieke L. F. van Lieshout^1,2^*, Floris P. de Lange^1,x^, & Roshan Cools^1,2,x^

Affiliations:

**^1^** Donders Institute for Brain, Cognition and Behaviour, Radboud University, P.O. Box 9101, 6500 HB Nijmegen, The Netherlands.

^2^ Department of Psychiatry, Radboud University Medical Centre, P.O. Box 9101, 6500 HB Nijmegen, The Netherlands.

Corresponding author (*):

Lieke L. F. van Lieshout

Donders Institute for Brain, Cognition and Behaviour, Radboud University

P.O. Box 9101

6500 HB Nijmegen

The Netherlands

E-mail: l.vanlieshout@donders.ru.nl

^x^ Both authors contributed equally to this work.

**Supplement 1: Analyses using repeated measures ANOVAs**

In addition to the results reported in the main text, the data of Experiment 1 and 2 were analyzed repeated measures ANOVAs in SPSS (RRID:SCR_002865). To this end, we divided the values of outcome uncertainty into “low outcome uncertainty” and “high outcome uncertainty”, such that approximately 50% of the trials were indicated as being low outcome uncertainty (outcome uncertainty <= 25) and approximately 50% as high outcome uncertainty (outcome uncertainty > 25). Additionally, we divided the values of absolute expected value into “low expected value” (expected value (absolute) < 50) and “high expected value” (expected value (absolute) > 50). Note that the trials with absolute expected value = 50 were omitted from the analyses. This was done because the values of absolute expected value are perfectly centered around expected value (absolute) = 50, precluding us to classify these trials as being either low or high expected value.

First, as main analyses we performed 2 (outcome valence: gain, loss) x 2 (outcome presentation: yes, no) x 2 (outcome uncertainty: low, high) x 2 (expected value (absolute): low, high) repeated measures ANOVAs with outcome valence (gain/loss), outcome presentation (yes/no), outcome uncertainty (low/high) and absolute expected value (low/ high) as within-subject factors. The dependent variable was either mean happiness as indicated by the happiness ratings (Experiment 1) or mean curiosity as indicated by the curiosity ratings (Experiment 2). Since not all participants used the full range of happiness or curiosity ratings, we z-scored the ratings per participant and calculated a mean of these z-scores per condition for each participant.

If the interaction effects between “outcome valence (gain/loss)” and either “outcome presentation (yes/no)”, “outcome uncertainty” or “expected value (absolute)” were significant in the main analysis, we ran additional 2 (outcome presentation: yes, no) x 2 (outcome uncertainty: low, high) x 2 (expected value (absolute): low, high) repeated measures ANOVAs on the gain and loss trials separately. If the interaction effects between “outcome presentation (yes/no)” and either “outcome uncertainty” or “expected value (absolute)” were significant in one of these additional analyses, we ran 2 (outcome uncertainty: low, high) x 2 (expected value (absolute): low, high) repeated measures ANOVAs on the trials in which the outcome was presented and not presented of the gain and loss trials separately.

Similarly, if the interaction effects between “outcome presentation (yes/no)” and either “outcome valence (gain/loss)”, “outcome uncertainty” or “expected value (absolute)” were significant in the main analysis, we ran 2 (outcome valence: gain, loss) x 2 (outcome uncertainty: low, high) x 2 (expected value (absolute): low, high) repeated measures ANOVAs on the trials in which the outcome was presented and not presented separately. If the interaction effects between “outcome valence (gain/loss)” and either “outcome uncertainty” or “expected value (absolute)” were significant in one of these additional analyses, we ran 2 (outcome uncertainty: low, high) x 2 (expected value (absolute): low, high) repeated measures ANOVAs on the gain and loss trials of outcome presented and not presented trials separately.

In JASP (RRID:SCR_015823), we performed the Bayesian equivalent of the repeated measures ANOVAs reported above. We used the default Cauchy prior to compute Bayes Factors (BF) for each effect. For interpretability in analyses with multiple factors, we used model averaging across matched models to get a single BF for each effect in the repeated measures ANOVA. This BF reflects the change from prior to posterior inclusion odds. It can intuitively be understood as the amount of evidence that the data gives for including an experimental factor in a model. The BF will converge to zero when the factor should not be included in the model, or to infinity when the factor should be included in the model. Values close to one indicate that there is not enough evidence for either conclusion.

*Experiment 1:*

As expected, participants were happier about gain lotteries compared with loss lotteries (Figure 2A; **RMA:** F(1,33) = 1955.1, *p* = 5.94e-31, ƞ_p_^2^ = .98, BF_incl_ = ∞). Happiness ratings were not different between blocks in which people would either see or not see the outcome of the lottery (**RMA:** F(1,33) = .07, *p* = .80, ƞ_p_^2^ =.002, BF_incl_ = .09). There was no main effect of absolute expected value (**RMA:** F(1,33) = .006, *p* = .94, ƞ_p_^2^ = 1.72e-04, BF_incl_ = .09), but there was an interaction between absolute expected value and outcome valence (**RMA:** F(1,33) = 450.9, *p* = 8.16e-21, ƞ_p_^2^ = .93, BF_incl_ = 2.67e+193). This interaction was due to participants being happier with higher gains compared with lower gains (**RMA:** F(1,33) = 466.9, *p* = 4.75e-21, ƞ_p_^2^ = .93, BF_incl_ = 3.36e+84), and with lower losses compared with higher losses (**RMA:** F(1,33) = 365.5, *p* = 2.02e-19, ƞ_p_^2^ = .92, BF_incl_ = 1.23e+94).

Furthermore, there was a main effect of outcome uncertainty (**RMA:** F(1,33) = 8.2, *p* = .007, ƞ_p_^2^ = .20, BF_incl_ = 1.43), such that participants were happier about trials with low compared with high outcome uncertainty. There was no interaction between outcome valence and outcome uncertainty when analyzing the data with a repeated measures ANOVA (**RMA:** F(1,33) = 1.2, *p* = .28, ƞ_p_^2^ = .04, BF_incl_ = .18), whereas we found a small but significant effect when analyzing the data with the BRMS package in R (see *Results*). However, when we did analyze the data of the gain and loss trials separately, we found a significant effect of outcome uncertainty on happiness in the loss trials (**RMA:** F(1,33) = 13.8, *p* = 7.5e-4, ƞ_p_^2^ = .30, BF_incl_ = 12.35), but not in the gain trials (**RMA:** F(1,33) = 1.6, *p* = .21, ƞ_p_^2^ = .05, BF_incl_ = .35). Therefore, we conclude that the negative effect of outcome uncertainty on happiness is particularly present in the loss trials (Figure 3a), although we acknowledge that the evidence for differential effects of outcome uncertainty between gain and loss trials is weak. There were no interaction effects between outcome valence, outcome uncertainty, absolute expected value and outcome presentation (yes/no), indicating that none of the reported effects depended on outcome presentation.

**­­­***Experiment 2:*

Participants were more curious about gain compared with loss lotteries (Figure 2B; **RMA:** F(1,33) = 19.6, *p­­* = 9.78e-05, ƞ_p_^2^ = .37, BF_incl_ = 1.58e+26). However, curiosity ratings were not different between blocks in which people would either see or not see the outcome of the lottery (**RMA**: F(1,33) = .10, *p* = .75, ƞ_p_^2^ = .003, BF_incl_ = .11). We found a robust effect of outcome uncertainty, such that participants were more curious about lotteries with higher compared with lower outcome uncertainty (**RMA:** F(1,33) = 80.0, *p* = 2.45e-10, ƞ_p_^2^ = .71, BF_incl_ = 5.06e+49). There was no interaction between outcome valence and outcome uncertainty (**RMA:** F(1,33) = 0.22, *p* = .64, ƞ_p_^2^ = .007, BF_incl_ =.13), indicating that this was the case for gain as well as for loss trials (Figure 3B).

We found a significant interaction between outcome valence and absolute expected value (**RMA:** F(1,33) = 5.08, *p* = .031, ƞ_p_^2^ = .13, BF_incl_ =.42). This interaction was due to participants being more curious for high compared with low absolute expected value in gain trials (**RMA:** F(1,33) = 10.5, *p* = .003, ƞ_p_^2^ = .24, BF_incl_ = 7.56), but not in loss trials (**RMA:** F(1,33) = 0.078, *p* = .78, ƞ_p_^2^ = .002, BF_incl_ = .13). Also in Experiment 2, there were no interaction effects between outcome valence, outcome uncertainty, absolute expected value and outcome presentation (yes/no), indicating that none of the reported effects was dependent on outcome presentation.

**Supplementary Figures**


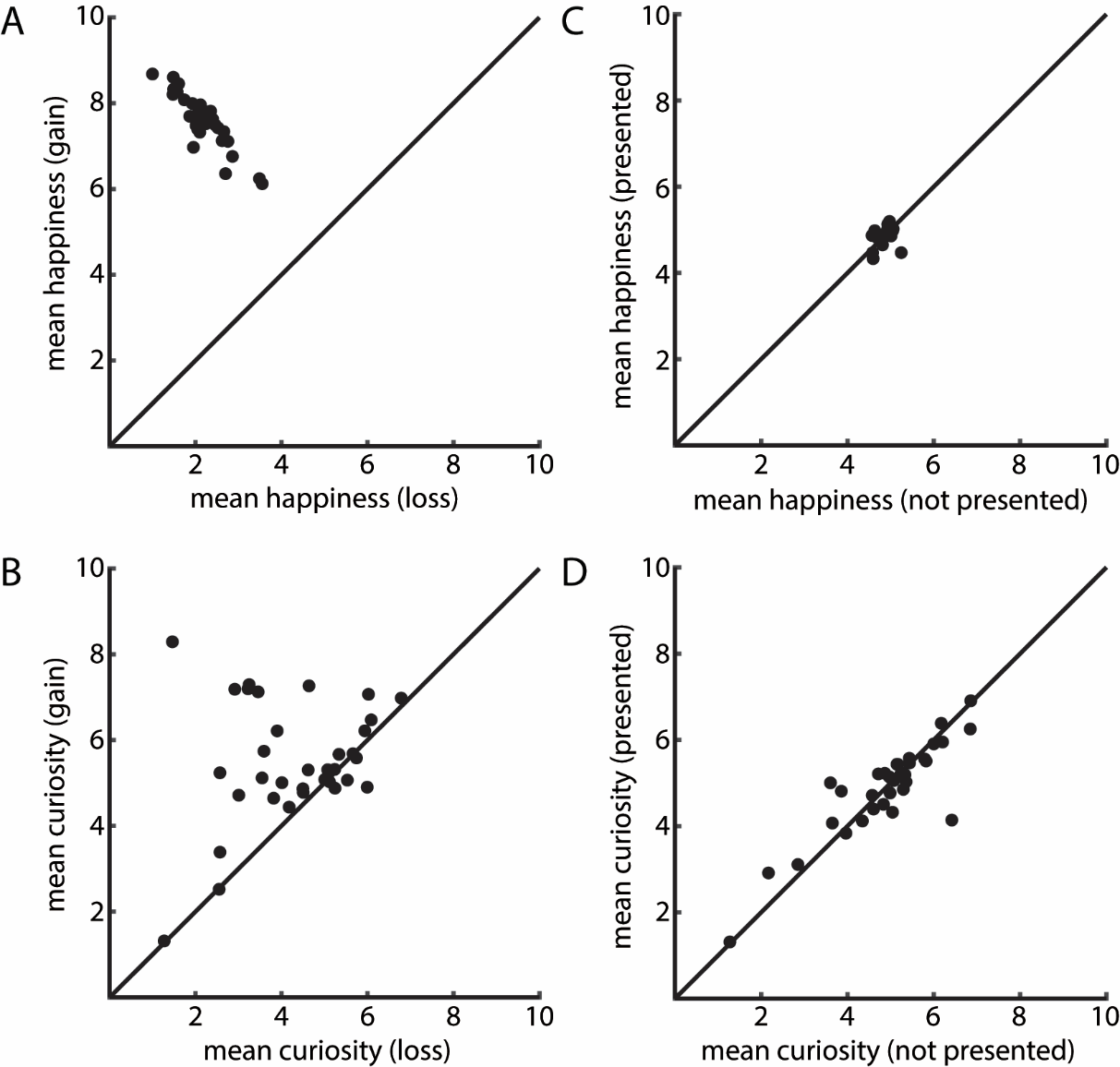


**Supplementary Figure 1: Individual variability in the effect of outcome valence and outcome presentation on happiness / curiosity**

**A.** Panel A depicts individual data points representing effects of outcome valence on happiness. The x-axis depicts mean happiness for loss lotteries and the y-axis mean happiness for gain lotteries. One dot depicts one participant. All dots are above the diagonal, indicating that all participants were happier when gain compared with loss lotteries would be played.

**B.** Panel B depicts individual data points representing effects of outcome valence on curiosity. Other conventions are as for panel A. The great majority of the dots are above the diagonal, indicating that most participants are more curious about the outcome of gain compared with loss lotteries.

**C.** Panel C depicts individual data points representing effects of outcome presentation on happiness. The x-axis depicts mean happiness for lotteries in which the outcome would not be presented and the y-axis mean happiness for lotteries in which the outcome would be presented. One dot depicts one participant. All dots are clustered on the diagonal, indicating that there was no effect of outcome presentation on happiness.

**D.** Panel D depicts individual data points representing effects of outcome presentation on curiosity. Other conventions are as for panel C. All dots are clustered around the diagonal, indicating that there was no effect of outcome presentation on curiosity.


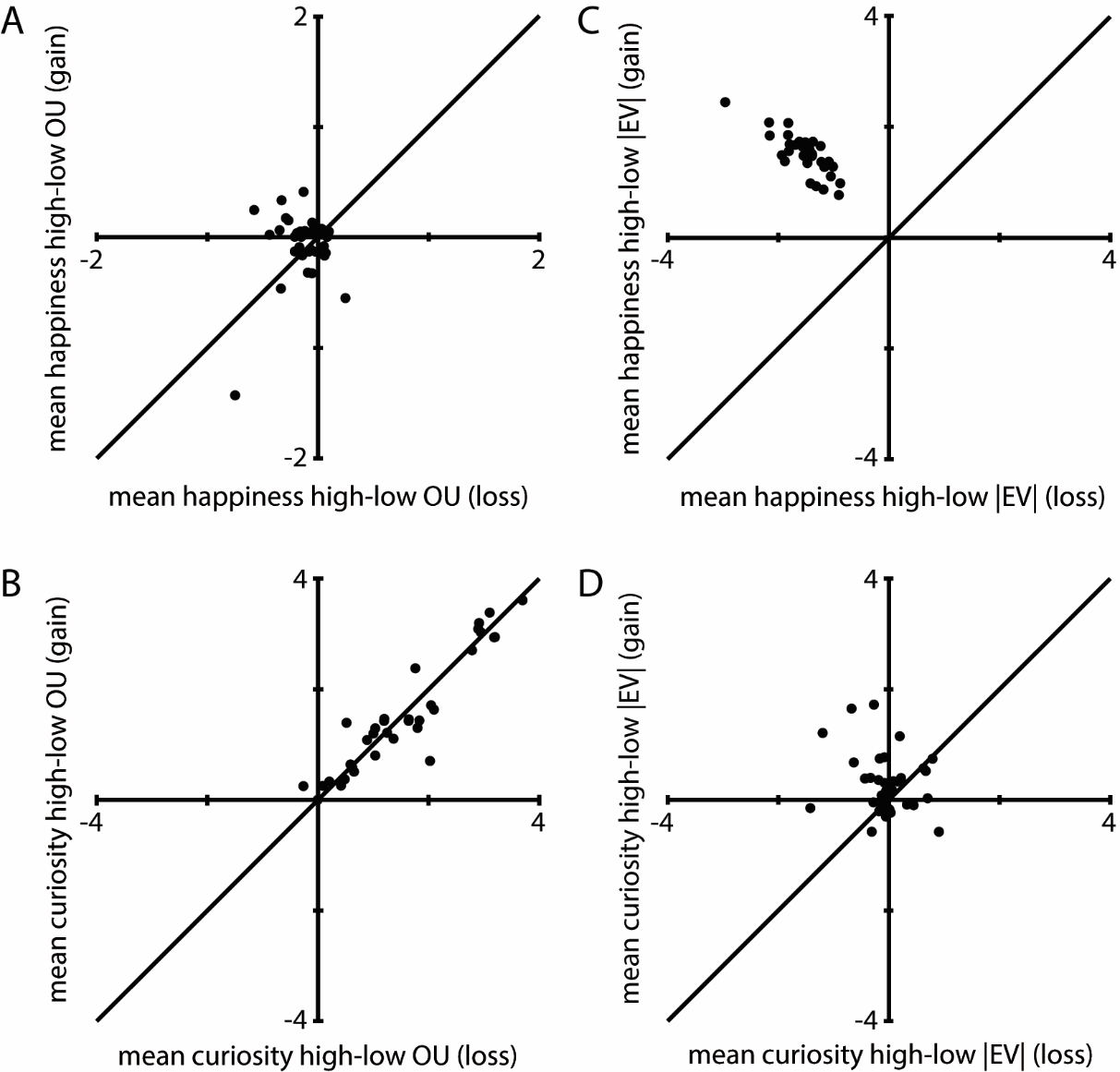


**Supplementary Figure 2. Individual variability in the effects of outcome uncertainty and absolute expected value on happiness (A,C) and curiosity (B,D) as a function of outcome valence**

**A.** Panel A depicts individual data points representing effects of outcome uncertainty on happiness, as a function of outcome valence (x-axis: losses, y-axis: gains). The x-axis depicts the mean happiness for high minus low outcome uncertainty in loss trials, with positive and negative values indicating a positive or negative relationship between outcome uncertainty and happiness in loss trials respectively. The y-axis depicts the mean happiness for high minus low outcome uncertainty in gain trials, with positive and negative values indicating a positive or negative relationship between outcome uncertainty and happiness in gain trials respectively. Every dot depicts one participant. Most participants have negative values on the x-axis, indicating that they are happier about low compared with high outcome uncertainty in loss trials. Participants vary in the extent to which extent they are sensitive to high compared with low outcome uncertainty in the win trials, as shown by the positive as well as negative values on the y-axis. However, most participants show a negative value on the y-axis, explaining the overall negative relationship between outcome uncertainty and happiness for both gains and losses.

**B.** Panel B has the same conventions as panel a, but this time for curiosity instead of happiness. Almost all participants show positive values on the x-axis (loss trials) as well as on the y-axis (gain trials), indicating that they are more curious about high compared with low outcome uncertainty in both gain and loss trials.

**C.** Panel C depicts individual data points representing effects of outcome uncertainty on happiness, as a function of outcome valence (x-axis: losses, y-axis: gains). The x-axis depicts the mean happiness for high minus low absolute expected value in loss trials, with positive and negative values indicating a positive or negative relationship between absolute expected value and happiness in loss trials respectively. The y-axis depicts the mean happiness for high minus low absolute expected value in gain trials, with positive and negative values indicating a positive or negative relationship between absolute expected value and happiness in gain trials respectively. Every dot depicts one participant. All dots are in the upper left quadrant, indicating that all participants are happier about high compared with low expected value in the gain trials (positive values on the y-axis), and happier about low compared with high absolute expected value in the loss trials (negative values on the x-axis).

**D.** Panel D has the same conventions as panel c but this time for curiosity instead of happiness. Most participants have positive values on the y-axis, indicating that they are more curious about high compared with low absolute expected value in gain trials. However, participants vary in the extent to whether they are more curious about high compared with low absolute expected value in loss trials, indicated by negative as well as positive values on the x-axis.
